# Up-regulation of ANKDR49, a poor prognostic factor, regulates cell proliferation of gliomas

**DOI:** 10.1042/BSR20170800

**Published:** 2017-08-04

**Authors:** Chunyan Hao, Hubin Duan, Hao Li, Mingyang Pei, Yueting Liu, Yimin Fan, Ce Zhang

**Affiliations:** 1 Department of Geriatrics, First Clinical Medical College of Shanxi Medical University, 85 Jie Fang South Road, Taiyuan 030001, Shanxi Province, People’s Republic of China; 2 Department of Neurosurgery, First Clinical Medical College of Shanxi Medical University, 85 Jie Fang South Road, Taiyuan 030001, Shanxi Province, People’s Republic of China

**Keywords:** ANKRD49, apoptosis, cell cycle, malignant glioma, proliferation

## Abstract

The Ankyrin repeat domain 49 (ANKRD49) is an evolutionarily conserved protein, which is related to mediate protein–protein interaction. However, the function of ANKRD49 in human glioma remains elusive. Mining through The Cancer Genome Atlas (TCGA) database, we found that the expression of ANKRD49 was increased in glioma tissues and that high expression of ANKRD49 was strongly associated with high disease grade and poor overall survival. To investigate the role of ANKRD49 in malignant glioma, lentivirus expressing shRNA targetting ANKRD49 was constructed in U251 and U87 malignant glioma cells. We demonstrated that ANKRD49 knockdown reduced the proliferation rate of U251 and U87 cells. Further mechanism analysis indicated that depletion of ANKRD49 led to the cell-cycle arrest and induced apoptosis in U251 and U87 cells. ANKRD49 knockdown also changed the expression of key effectors that are involved in stress response, cell cycle, and apoptosis, including p-HSP27 (heat shock protein 27), p-Smad2 (SMAD family member 2), p-p53, p-p38, p-MAPK (mitogen-activated protein kinase), p-SAPK/JNK (stress-activated protein kinase/c-jun n-terminal kinase), cleveagated Caspase-7, p-Chk1 (checkpoint kinase 1), and p-eIF2a (eukaryotic translation initiation factor 2a). Taken together, our findings implicate that ANKRD49 promotes the proliferation of human malignant glioma cells. ANKRD49 maybe an attractive target for malignant glioma therapy.

## Introduction

Glioma is one of the most common tumors in the brain or spine that arises from the glial cells [1]. Epidemiological studies have revealed that glioma makes up approximately 30% of all brain and central nervous system tumors and 80% of all malignant brain tumors [2]. According to the pathologic evaluation of the tumor, gliomas are categorized into two main types: low-grade gliomas (LGG) (World Health Organization, WHO grade II) and high-grade (WHO grade III–IV) gliomas [3]. Glioblastoma, also known as glioblastoma multiforme (GBM), has been considered as the most aggressive tumor in the brain, since the cells reproduce quickly and they are supported by a large network of blood vessels [4]. Besides, prognosis of high-grade gliomas is generally poor with an average survival less than 12 months after diagnosis [5]. However, gliomas are still rarely curable. Therefore, it is urgent and necessary to find effective therapies for gliomas [6]. Further understanding of the genetic and molecular mechanisms of gliomas will provide promising strategies for treatment of the diseases.

The ankyrin repeat domain 49 (ANKRD49) contains four ankyrin repeats, a motif of 30–34 amino acid residues that was first identified in the yeast Swi6p, septin 7 pseudogene 1 (*Cdc10p*), and Notch [7,8]. As the most widely abundant protein, ankyrin repeat proteins mediate protein–protein interaction. It is widely known that some of them are directly associated with the development of diverse human cancers [9]. For instance, the Notch protein, a key component of cell signaling pathways, can cause neurological disorder when the repeat domain is disrupted by mutations [10]. However, the function of ANKRD49 remains largely unknown. A recent work reported that ANKRD49 played an important role in spermatogenesis by enhancing the nuclear factor κB (NF-κB) pathway to augment autophagy in male germ GC-1 cells [11]. In addition, ANKRD49 is lowly expressed in rat lateral habenula in a depression model of responders [12]. In human patients of non-small-cell lung cancer, ANKRD49 serves as an invasion-associated gene and predicts patients’ survival [13]. However, the role of ANKRD49 in glioma remains largely unclear.

Here, in the present study, we found that the expression of ANKRD49 in human glioma is significantly increased in comparison with normal donors. High expression of ANKRD49 is correlated with high disease grade and poor overall survival in glioma patients. ANKRD49 knockdown impaired the proliferation of U251 and U87 malignant glioma cells. We also found that the depletion of ANKRD49 repressed cell cycle to enter M-phase and induced apoptosis in U251 and U87 cells. Our molecular analysis also confirmed that ANKDR49 altered the phosphorylation of some effectors related to stress response, apoptosis, and cell cycle, including p-HSP27 (heat shock protein 27), p-Smad2, p-p53, p-p38, p-MAPK (mitogen-activated protein kinase), p-stress-activated protein kinase/c-jun n-terminal kinase (SAPK/JNK), p-Chk1 (checkpoint kinase 1), p-eIF2a (eukaryotic translation initiation factor 2a), as well as the cleavage of Caspase-7.

## Materials and methods

### TCGA gene expression data

Transcriptome expression datasets and the corresponding clinical information of *ANKRD49* gene were downloaded from the website of The Cancer Genome Atlas (TCGA) (http://cancergenome.nih.gov). A total of 558 samples, which contained transcriptional expression data of 548 tumor tissues and 10 normal tissues, were available for this analysis.

### Cell culture

Human malignant glioma cell lines U251 and U87 were cultured in Dulbecco’s modified Eagle’s medium (DMEM) (Invitrogen), supplemented with 10% FBS (Corning) and 1% penicillin and streptomycin solution (Corning). Cells were cultured at 37°C with 5% CO_2_.

### Total mRNA isolation and quantitative real-time PCR

Total RNA was isolated from indicated cells using TRIzol reagent (Invitrogen) according to the manufacturer’s instructions. Moloney murine leukemia virus (M-MLV) reverse transcriptase (Promega) was used for cDNA synthesized. Quantitative real-time PCR was used to analyze *ANKRD49* gene expression using synergy brands (SYBR) master mixture (Takara). The PCR primers used were as follows: ANKRD49 forward, 5′-GGTACTCAAAGTCTTTGGGTAGG-3′ and reverse: 5′-AGAAGCAATCTGCTTGGGTCT-3′; and glyceraldehyde-3-phosphate dehydrogenase (GAPDH), forward: 5′-TGACTTCAACAGCGACACCCA-3′ and reverse: 5′-CACCCTGTTGCTGTAGCCAAA-3′. The relative ANKRD49 expression was normalized to GAPDH, and data analysis was conducted using the comparative cycle threshold (*C*_T_) method.

### Western blot

Total protein was isolated from indicated cells using lysis buffer (Beyotime) and concentration was determined by BCA Protein Assay Kit (Beyotime). Twenty micrograms of total protein, mixed with 2× loading buffer, was separated on an SDS/PAGE (12% gel) and transferred on to PVDF membrane (Millipore, U.S.A.). The membranes were blocked with 5% skim milk for 1 h at room temperature and immunoblotted with primary antibodies at 4°C overnight. Antibody against ANKRD49 was purchased from Abcam. Antibodies against GAPDH, p-ERK, ERK were from Santa Cruz. Antibodies against Survivin, p-HSP27, HSP27, p-Smad2, Smad2, p-Chk1, and Chk1 were obtained from Cell Signaling Technology.

### Packaging of lentivirus

The lentivirus system is a composed shANKRD49 vector (pGCSIL-GFP, stably expressed shRNA fused with a GFP marker) and two helper vectors (pHelper1.0: gag/pol element and Helper2.0: VSVG element). shRNA targetting human ANKRD49 (5′-AGGGATGAAGATGAGTATA-3′) and the control shRNA used as negative control (5′-TTCTCCGAACGTGTCACGT-3′) were designed, synthesized, and cloned into the pGCSIL-GFP vector by GeneChem (Shanghai, China). The three vectors were mixed and transfected into 293T cells with Lipofectamine™ 2000 (Invitrogen, Shanghai, China). After 48 h of transfection, viral supernatants were collected, centrifuged, and filtered through 0.45-µm PVDF membranes.

### High-content screening assay

U251 cells infected with shCtrl lentivirus or shANKRD49 lentivirus were seeded and cultured in 96-well plates for 5 days. Cell numbers in each well was determined using the ArrayScan™ high-content screening (HCS) software (Cellomics Inc) each day. The number and distribution of stained cells were identified and analyzed by the fluorescence imaging microscope. Images were acquired using appropriate filters with 20× objective and stored in a Microsoft structured query language (SQL) database.

### MTT assay

U251 and U87 cells infected with shCtrl lentivirus or shANKRD49 lentivirus were seeded in 96-well plates at an initial density of 2000 cells/well. Cell viability was measured at day 1, 2, 3, 4, and 5 respectively. Each well was added into MTT solution (5 mg/ml). After 4 h of incubation, supernatants from each well were removed and 100 μl of DMSO was added to solubilize the formazan salt. Ten minutes later, the optical density (OD) was measured at 490 nm by using a microplate reader.

### Cell cycle assay

Cell cycle progression was examined on a flow cytometer using Propidium Iodide (PI, Sigma–Aldrich) staining. U251 and U87 cells infected with shCtrl lentivirus or shANKRD49 lentivirus were seeded in six-well culture plates. Cell cycle was analyzed by PI staining. Then, PI absorbance was determined by fluorescence-activated cell sorting on flow cytometry.

### Apoptosis assay

Cell apoptosis assay was analyzed using annexin V-allophycocyanin (APC) Apoptosis Detection Kit (Ebioscience, U.S.A.) according to the manufacturer’s protocol. U251 and U87 cells infected with shCtrl lentivirus or shANKRD49 lentivirus were harvested, washed with PBS, and resuspended using staining buffer at a final density of 1 × 10^6^ ml. Five microliters of annexin V-APC was added into 100 μl of the above cell suspension. Then, the mix was incubated at room temperature for 15 min, finally subjected to flow cytometry (FACSCalibur, Becton-Dickinson, U.S.A.).

### Stress and apoptosis signaling assay

To address the signaling pathway involved in the phenotype induced by ANKRD49, we detected the modifications in a set of cellular proteins playing a well-understood role in cell proliferation and apoptosis using PathScan® Stress and Apoptosis Signaling Array Kit (Cell Signaling Technology, #12856). U251 or U87 cell lysate was prepared and detected according to the protocol provided by CST.

### Statistical analysis

The statistical analyses were performed using SPSS 16.0 software (SPSS, Inc., Chicago, IL, U.S.A.). The data shown are presented as the mean ± S.E.M. of at least three independent repeats if no any other statements. Students’ *t* tests were used to analyze the differences between the two groups. One-way ANOVA analysis was applied for analyzing the difference for more than two groups. A probability value of less than 0.05 was considered significant.

## Results

### ANKRD49 is highly expressed in glioma and significantly correlated with glioma grade and survival

**Figure 1:**
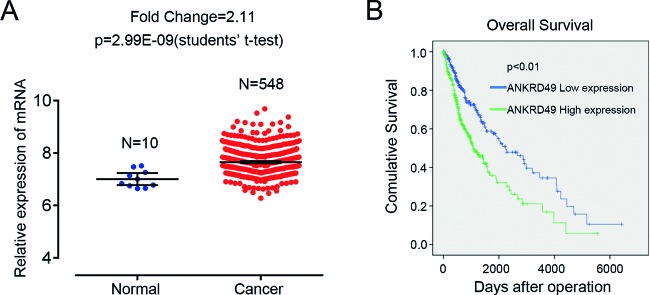
ANKRD49 is overexpressed in gliomas and its expression level is correlated with overall survival (**A**) Relative expression of ANKRD49 in gliomas and normal tissues of the patients from TCGA database (fold change =2.11, *P*=2.99E-09). (**B**) Kaplan–Meier overall survival curves according to *ANKRD49* mRNA level in glioma patients. ANKRD49 expression in gliomas correlates with overall survival.

Through reanalyzing RNA sequencing data of glioma-related datasets in TCGA database, we found that the expression of ANKRD49 was predominantly higher in glioma samples compared with matched normal samples (Figure 1A) (fold change =2.11, *P*=2.99E-09). To further explore the relationship between ANKRD49 and malignant glioma, we performed a clinical research in TCGA to study the expression pattern of ANKRD49 in LGG and GBM, two different malignant levels of glioma. In contrast with LGG, most GBM cases expressed high level of ANKRD49 (Table 1, *P*<0.001), indicating that the expression of ANKRD49 is positively related to the malignant level of glioma. Additionally, we investigated the association between ANKRD49 expression and patients’ survival. As shown in Figure 1B, the 5-year overall survival of patients with highly expressed ANKDR49 was distinctively shorter than that of patients with lowly expressed ANKDR49. Therefore, we concluded that ANKRD49 is overexpressed in human glioma tissues, and high expression of ANKRD49 is significantly associated with high disease grade and poor survival.

**Table 1:** Analysis for ANKRD49 between LGG and GBM in TCGA data (Mann–Whitney U-test)

		ANKRD49		Total	*P*-value
		Low expression, number	High expression, number		
Variables	LGG	305	210	515	
	GBM	29	123	152	0.000
Total		334	333	667	

### ANKRD49 expression is efficiently reduced by lentiviral-mediated shRNA in human glioma cell lines

**Figure 2:**
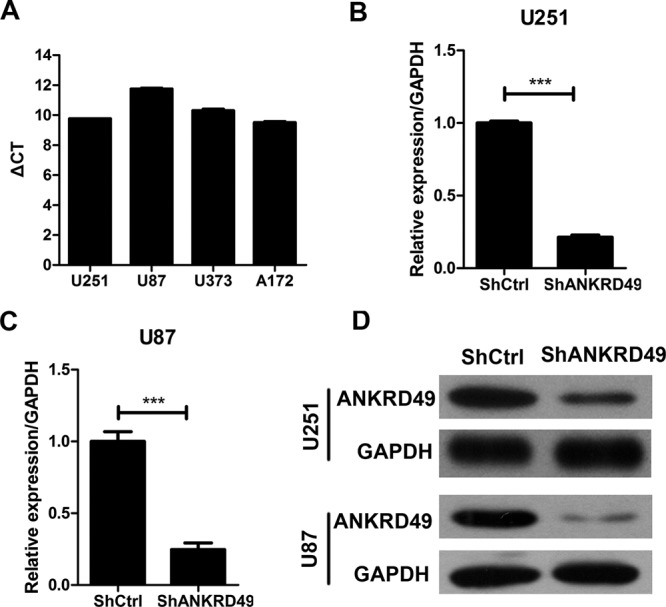
ANKRD49 expression and knockdown in human glioma cells (**A**) Expression of *ANKRD49* mRNA was measured by q-PCR in four human glioma cell lines: U251, U87, U373, and A172. (**B**,**C**) Quantitative RT-PCR analysis revealed that ANKRD49 expression was efficiently knocked down in the U251 (B) or U87 (C) cells; ***P<0.001. (**D**) Western blot analysis revealed that ANKRD49 expression was efficiently knocked down in the U251 or U87 cells. GAPDH served as an internal control.

Remarkable correlation between ANKRD49 and malignant glioma suggests that ANKRD49 may be involved in malignant glioma development and progression. As expected, ANKRD49 was found to be expressed in four human glioma cell lines (Figure 2A). To examine the causal role of ANKRD49 in malignant glioma, lentivirus-mediated shRNA strategy was employed to knockdown ANKRD49 in U251 and U87 cells. Then, quantitative real-time PCR (qRT-PCR) and Western blot were performed to determine ANKRD49 expression at both mRNA and protein level. As shown in Figure 2B, endogenous ANKRD49 was significantly reduced in the shANKRD49 U251 cells compared with the shCtrl cells (*P*<0.001). In addition, ANKRD49 was obviously silenced in U87 cells expressing shANKRD49 lentivirus compared with U87 cells expressing shCtrl (Figure 2C). Western blot results were consistent with qRT-PCR results (Figure 2D). Taken together, lentiviral-mediated shRNA strategy could efficiently inhibit ANKRD49 expression both at mRNA and protein levels.

### Cell proliferation is impaired by ANKRD49 knockdown in U251 and U87 cells

**Figure 3:**
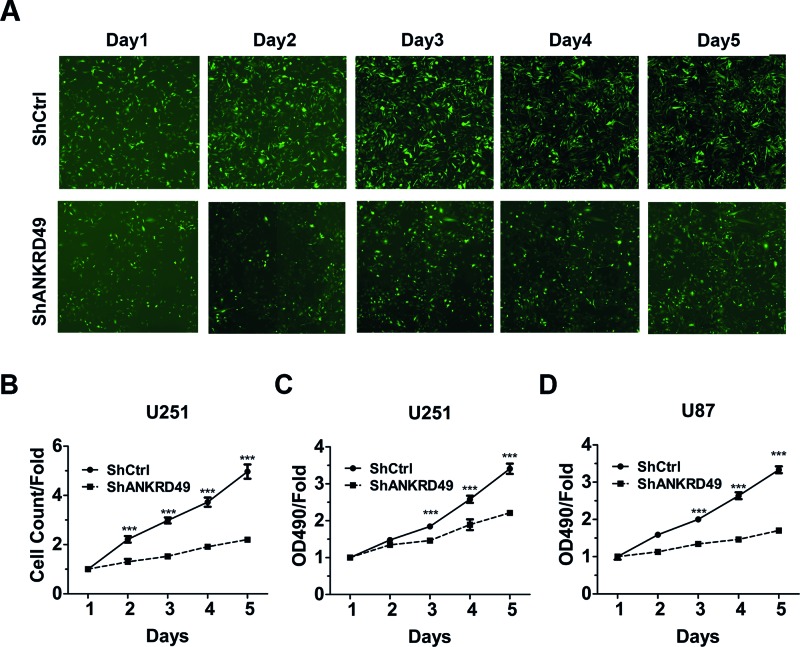
Knockdown of ANKRD49 inhibited the proliferation of U251 and U87 cells (**A**) Representative images of U251 cells infected with shCtrl (top) and shANKRD49 (bottom) via multipara metric HCS everyday for 5 days. (**B**) ANKRD49 knockdown represses U251 cell proliferation. U251 cells infected with shCtrl and shANKRD49, and cell number was analyzed everyday by HCS; ***P<0.001. (**C**) ANKRD49 knockdown inhibits the U251 cells proliferation that was determined by MTT assay; ***P<0.001. (**D**) ANKRD49 knockdown blunts the U87 cells proliferation that was determined by MTT assay; ***P<0.001.

To examine whether ANKRD49 contributes to the development of human malignant glioma, two different assays were employed to evaluate cell proliferation. First, we monitored the cell number of U251 cells everyday for 5 straight days. Obvious cell proliferation impairment was observed in U251 cells from the second day in the shANKRD49 group as compared with the shCtrl group (Figure 3A,B). Furthermore, the cell proliferation status of ANKRD49 knockdown on U251 cells was also tested by MTT assay. Compared with shCtrl U251 cells, the proliferative rate of U251 cells with ANKRD49 knockdown was significantly decreased from the third day (Figure 3C). Besides, we used MTT assay to test the effect of ANKRD49 silencing on U87 cells. As expected, ANKRD49 knockdown blunted the proliferation rate of U87 cells (Figure 3D). These results suggested that ANKRD49 is indispensable for U251 and U87 cell proliferation.

### Cell cycle arrest is induced by knockdown of ANKRD49

**Figure 4:**
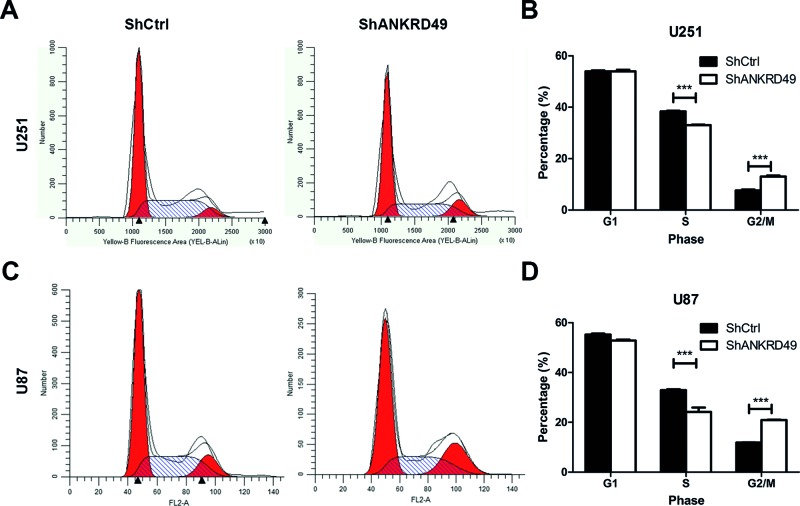
Knockdown of ANKRD49 regulates cell cycle of U251 cells (**A**) U251 cells were infected with shCtrl or shANKRD49 lentivirus for 72 h and then flow cytometry was performed to analyze the cell cycle. (**B**) Quantitation of cell cycle phase of U251 cells expressing shCtrl or shANKRD49 lentivirus; ***P<0.001. (**C**) U87 cells were infected with shCtrl or shANKRD49 lentivirus for 72 h and cell cycle was analyzed by flow cytometry. (**D**) Quantitation of cell cycle phase of U87 cells expressing shCtrl or shANKRD49 lentivirus; ***P<0.001.

As a common characteristic of tumors, uncontrolled cell cycle process permits persistent cell proliferation. To further explore the mechanisms underlying the inhibition of cell growth by ANKRD49 knockdown, flow cytometry plus PI staining was performed to determine whether ANKRD49 knockdown could impair cell cycle process. The cell-cycle distribution of U251 cells after ANKRD49 knockdown was shown in Figure 4A. We found that ANKRD49-knockdown cells were blocked at the G_2_-/M-phase because the cell percentage in the G_2_-/M-phase of cells with ANKRD49 knockdown was markedly increased compared with control cells (*P*<0.001). The cell populations of S-phase were concomitantly decreased following ANKRD49 knockdown (Figure 4B). Similar results were also observed in U87 cells (Figure 4C,D). These findings indicated that ANKRD49 is essential for G_2_–M transition in human glioma cell lines U251 and U87.

### Apoptosis of U251 cells is promoted by ANKRD49 knockdown

**Figure 5:**
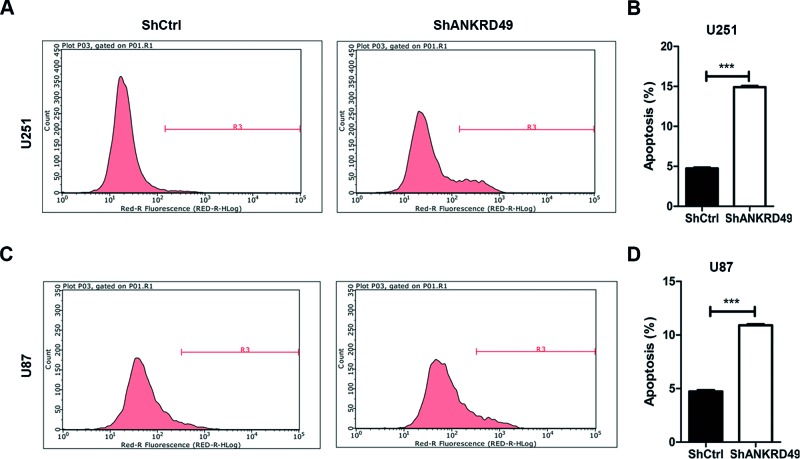
ANKRD49 knockdown induces apoptosis in U251 cells (**A**) U251 cells were infected with shCtrl or shANKRD49 for 72 h and then cell apoptosis was determined by annexin V-APC staining and flow cytometry. (**B**) Quantitation of U251 cell apoptosis induced by knockdown of ANKRD49 as shown in (A); ***P<0.001. (**C**) U87 cells were infected shCtrl or shANKRD49 for 72 h and then cell annexin V-APC staining and flow cytometry were performed to determine apoptosis. (**D**) Quantitation of U87 cell apoptosis induced by knockdown of ANKRD49 as shown in (**C**); ***P<0.001.

As cell cycle arrest may reflect the alterations of apoptosis, we next evaluated the effect of ANKRD49 knockdown on apoptosis of U251 and U87 cells by annexin V-APC assay plus flow cytometry. Apoptosis was detected in 1.85% cells infected with lentivirus expressing shRNA and in 14.91% cells infected with lentivirus expressing shANKRD49 (Figure 5A). Quantitative results indicated that apoptosis was significantly increased after ANKRD49 knockdown in U251 cells (Figure 5B). In addition, ANKRD49 knockdown also resulted in increased apoptosis of U87 cells (Figure 5C,D). Therefore, ANKRD49 knockdown promotes the apoptosis of glioma cells.

### ANKRD49 knockdown regulates stress response, cell cycle, and apoptotic effectors in U251 cells

**Figure 6:**
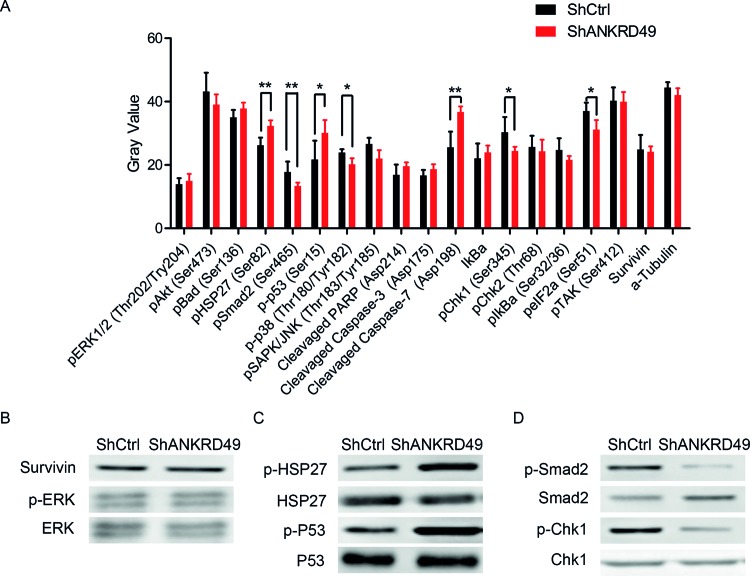
Alterations of protein modification induced by ANKRD49 knockdown are detected through stress and apoptosis signaling assay and Western blot (**A**) The post-translational modification and total protein level of 18 important signaling proteins (13 proteins for phosphorylation, 2 ptoteins for expression level, and 3 proteins for cleavage) was monitored by array hybridization. α-tubulin is used as an internal control; *P<0.05 and **P<0.01. (**B**–**D**) Western blot analysis of shANKRD49 or shCtrl U251 cells with antibodies against survivin, p-ERK, ERK (B), and p-HSP27, HSP27, p-P53, p53 (C), and p-Smad2, Smad2, p-Chk1, Chk1 (D).

To explore the underlying signaling pathways mediated by ANKRD49 in malignant glioma cells, PathScan® Stress and Apoptosis Signaling Array Kit was utilized. The changed signaling pathways were involved in stress response, cell cycle, and apoptosis, including MAPK/ERK cascade, p38 and JNK MAPKs, Stat1 and Stat3, Akt, p38-MAPK, HSP27, p53, and caspase-3. The alterations of modifications occurred in these signaling proteins were quantitated and shown in Figure 6A. To our surprise, down-regulation of ANKRD49 in U251 cells resulted in a series of phosphorylation alteration, including p-HSP27 (Ser^82^), p-Smad2 (Ser^465^), p-p53 (Ser^15^), p-p38 MAPK (Thr^180^/Try^182^), p-SAPK/JNK (Thr^183^/Try^185^), p-Chk1 (Ser^345^), p-eIF2a (Ser^51^), as well as the cleavage of Caspase-7 (Figure 6A). To further verify these altered proteins, immunoblotting assay was performed. As expected, survivin and p-ERK remained unchanged after ANKRD49 knockdown (Figure 6B). Additionally, the levels of p-HSP27 and p-p53 were increased, while p-HSP27 (Ser^82^) and p-Chk1 (Ser^345^) were decreased after ANKRD49 knockdown in U251 cells (Figure 6C,D). These data suggest that the involvement of ANKRD49 in U251 may be partly via the modulation of related signaling proteins.

## Discussion

In the present study, we investigated the clinical implication of ANKRD49 in glioma patients and the function of ANKRD49 in human malignant glioma cells U251 and U87. We showed that ANKRD49 expression is positively correlated to glioma malignancy and accordingly has adverse impacts on overall survival rate. These results provide compelling evidence that ANKRD49 is crucial to malignant glioma progression and its expression level is an important signature to predict prognosis. To gain deep insight into the functional role of ANKRD49 in malignant glioma progress, we utilized lentivirus-mediated shRNA to knockdown ANKRD49 expression in malignant glioma cells U251 and U87. Using this loss-of-function strategy, we demonstrated that ANKRD49 knockdown inhibited the proliferation rate of U251 and U87 cells. Our mechanism study indicated that ANKRD49 knockdown repressed cell cycle to enter M-phase and induced apoptosis. Furthermore, our molecular analysis confirmed that ANKRD49 regulated modifications of effector proteins involved in stress response, cell cycle, and apoptosis.

Malignant glioma accounts for 80% of the total incidence of primary central nervous system tumor, which is essentially incurable [14,15]. This dismal clinical outcome makes glioma an urgent subject of cancer research. TCGA project provided a comprehensive view of the complicated landscape of gliomas [16,17], enabling us to explore the potential impact of ANKRD49 on glioma patients. Here, we showed that ANKRD49 expression is positively related to glioma grade and manifests a significant prognosis difference between glioma samples and non-glioma samples, suggesting that ANKRD49 may be a prognosis predictor. Similar results from a previous study showed that ANKRD49, as one of the four gene signature from NCI-60 cell line, had a strong prediction in non-small-cell lung cancer [13]. These results indicated that ANKRD49 could be a potential prognosis predictor of pan-cancer.

Using loss-of-function approach, we observed that ANKRD49 knockdown disrupted cell cycle progression and induced apoptosis, resulting in the repression of U251 cell proliferation. Recent study reported that ANKRD49 enhanced germ cell autophagy via NF-κB pathway in spermatogenesis [11]. These evidence imply that ANKRD49 may participate in various pathological processes. It is well established that there is a cross-talk between autophagy and apoptosis [18]. Normally, autophagy and apoptosis share common upstream signals, therefore resulting in similar cellular responses [19]. Therefore, whether ANKRD49 could regulate autophagy of malignant glioma cells needs further studies. However, IκBα, a crucial negative regulator in NF-κB pathway, has no change in the present study, implying that ANKRD49 may trigger different pathways in different cells.

Numerous studies have revealed a set of core signaling pathways commonly activated in gliomas: the p53 pathway, RB transcriptional corepressor 1 (RB) pathway, and receptor tyrosine kinase (RTK) pathway [17]. Alternations of all the three pathways help to fuel cell proliferation and enhance cell survival and allow the tumor cell to escape from cell-cycle checkpoints, senescence, and apoptosis [20]. Through PathScan® stress and apoptosis signaling analysis, we illustrated whether ANKRD49 regulated the three main pathways in U251 cells. First, the p53 pathway might be involved as ANKRD49 knockdown increased the phosphorylation abundance of p53 at Ser^15^ and the cleavage of caspase-7, two activated forms of proteins that are involved in regulating and executing apoptosis. Accordingly, the phosphorylated form of HSP27 was increased after ANKD49 knockdown, which opposes TNFα-induced apoptosis and results in cell cycle arrest [21,22]. Chk1 co-ordinates the DNA damage response and cell cycle checkpoint response [23,24]. In the present study, we also showed a decreased level of Chk1 phosphorylation, indicating that Chk1 may participate in the oncogenic function of ANKRD49 in malignant glioma cells. Furthermore, RTK/RAS/MAPK signaling pathway was significantly affected by ANKRD49. p38 is a class of MAPKs that are responsive to stress stimuli and is involved in cell differentiation, apoptosis, and autophagy [25,26]. Interestingly, p38 phosphorylation was decreased in malignant glioma cells expressing lenti-shANKRD49, which may partly contribute to ANKRD-induced apoptosis. In our study, the phosphorylation level of HSP27, Chk1, p53, and p38, as well as cleavage of caspase-7 was elevated in ANKRD49-silenced glioma cells, adding the evidence that depletion of ANKRD49 could suppress malignant glioma cell growth via the induction of cell-cycle arrest and apoptosis. However, further works are needed to elucidate how ANKRD49 regulates these possible pathways to modulate cell cycle, apoptosis, and cell proliferation.

In conclusion, we identify ANKRD49 as an oncogene in malignant gliomas. Our findings indicate that ANKRD49 promotes cell proliferation of U251 and U87 cells by controlling cell cycle and survival. Therefore, ANKRD49 may serve as a prognostic factor and a potential therapeutic target in human malignant glioma.

## Abbreviations

Akt: protein kinase B
APC: allophycocyanin
ANKRD49: ankyrin repeat domain 49
Chk1: checkpoint kinase 1
eIF2a: eukaryotic translation initiation factor 2a
ERK: extracellular signal-regulated kinase
GAPDH: glyceraldehyde-3-phosphate dehydrogenase
GBM: glioblastoma multiforme
HCS: high-content screening
HSP27: heat shock protein 27
LGG: low-grade glioma
MAPK: mitogen-activated protein kinase
M-MLV: Moloney murine leukemia virus
NF-κB: nuclear factor κB
PI: propidium iodide
qRT-PCR: quantitative real-time PCR
RTK: receptor tyrosine kinase
SAPK/JNK: stress-activated protein kinase/c-jun n-terminal kinase
Smad2: SMAD family member 2
STAT: signal transducer and activator of transcription
TCGA: The Cancer Genome Atlas
TNF: tumor necrosis factor
WHO: World Health Organization
